# Stress-induced tunneling nanotubes support treatment adaptation in prostate cancer

**DOI:** 10.1038/s41598-019-44346-5

**Published:** 2019-05-24

**Authors:** Alexander Kretschmer, Fan Zhang, Syam Prakash Somasekharan, Charan Tse, Lauren Leachman, Anna Gleave, Brian Li, Ivan Asmaro, Teresa Huang, Leszek Kotula, Poul H. Sorensen, Martin E. Gleave

**Affiliations:** 1 The Vancouver Prostate Centre, Department of Urological Sciences, University of British Columbia, 2775 Laurel Street, Vancouver, British Columbia V6H 3Z6 Canada; 2 Department of Urology, Biochemistry and Molecular Biology, Medicine SUNY Upstate Medical University, Syracuse, NY 13210 USA; 3 Department of Pathology and Laboratory Medicine, University of British Columbia, Vancouver, British Columbia V6H 3Z6 Canada

**Keywords:** Prostate, Prostate cancer

## Abstract

Tunneling nanotubes (TNTs) are actin-based membranous structures bridging distant cells for intercellular communication. We define roles for TNTs in stress adaptation and treatment resistance in prostate cancer (PCa). Androgen receptor (AR) blockade and metabolic stress induce TNTs, but not in normal prostatic epithelial or osteoblast cells. Co-culture assays reveal enhanced TNT formation between stressed and unstressed PCa cells as well as from stressed PCa to osteoblasts. Stress-induced chaperones clusterin and YB-1 localize within TNTs, are transported bi-directionally via TNTs and facilitate TNT formation in PI3K/AKT and Eps8-dependent manner. AR variants, induced by AR antagonism to mediate resistance to AR pathway inhibition, also enhance TNT production and rescue loss of clusterin- or YB-1-repressed TNT formation. TNT disruption sensitizes PCa to treatment-induced cell death. These data define a mechanistic network involving stress induction of chaperone and AR variants, PI3K/AKT signaling, actin remodeling and TNT-mediated intercellular communication that confer stress adaptative cell survival.

## Introduction

In advanced prostate cancer (PCa), androgen receptor (AR) pathway inhibition (ARPI) induces profound and sustained responses. However, progression to castration-resistant prostate cancer (CRPC) is inevitable and attributable to genomic and metabolic re-activation of the AR^1^ supported by context-dependent activation of stress response^2–4^, kinase and developmental pathways^5,6^. In addition to those induced by therapy, tumors are exposed to diverse stresses that are potentially lethal unless cells can acutely adapt to them. Cancer cells often coopt key homeostatic stress responses that contribute to cell survival and therapy resistance; indeed, the “stress phenotype” (eg. DNA damage, mitotic, metabolic, proteotoxic and oxidative stress) is considered a hallmark of cancer^7^.

We previously defined several stress adaptive mechanisms that promote treatment resistance in cancer, including roles for stress adaptor proteins like YB-1^8^, clusterin (CLU)^3^ and Hsp27^4^ in pro-survival pathways activation^9–11^. These stress chaperones play central roles in proteostasis through regulation of autophagy activation^9^, selective protein translation^12^, and stress granule formation^11^, as well as prosurvival signal transduction^13,14^ and transcriptional^15^ pathways. The tumor microenvironment is an important paradigm for cancer progression and therapeutic manipulation^16,17^, which includes stress-induced intercellular communication amongst cells in a heterogeneous tumor. In addition to cytokine-based signaling, exosomes, and connexin-based gap junctions^18^, F-actin-based membranous tunneling nanotubes (TNTs) have emerged as extracellular structures that can directly transport cellular cargo for intercellular communication^19,20^. First described in 2004^21^, TNTs are freely hovering channels with a diameter of up to 1 μm that can reach lengths up to several cell diameters^19,22–24^, and are involved in transport of cell organelles^25,26^, Ca^2+^, prions^27^, and HIV^28,29^. Various morphology of TNTs have been described in many cancer cell lines^30–33^ and patient tumors such as malignant pleural mesothelioma and lung adenocarcinoma^34^.

While emerging evidence highlight roles for stress adaptive changes in treatment resistance, mechanistic roles for TNTs in such plasticity are unclear. We set out to test the hypothesis that TNTs are induced by ARPI as an adaptive response involving stress chaperones like CLU and YB-1, to facilitate intercellular communication under stress conditions. In this study, we characterize the formation and features of TNTs in PCa cells after metabolic and ARPI stress and explore their roles in transport of stress-associated proteins involved in treatment resistance.

## Results

### AR blockade and metabolic stress induce TNT formation in PCa cells

**Figure 1:**
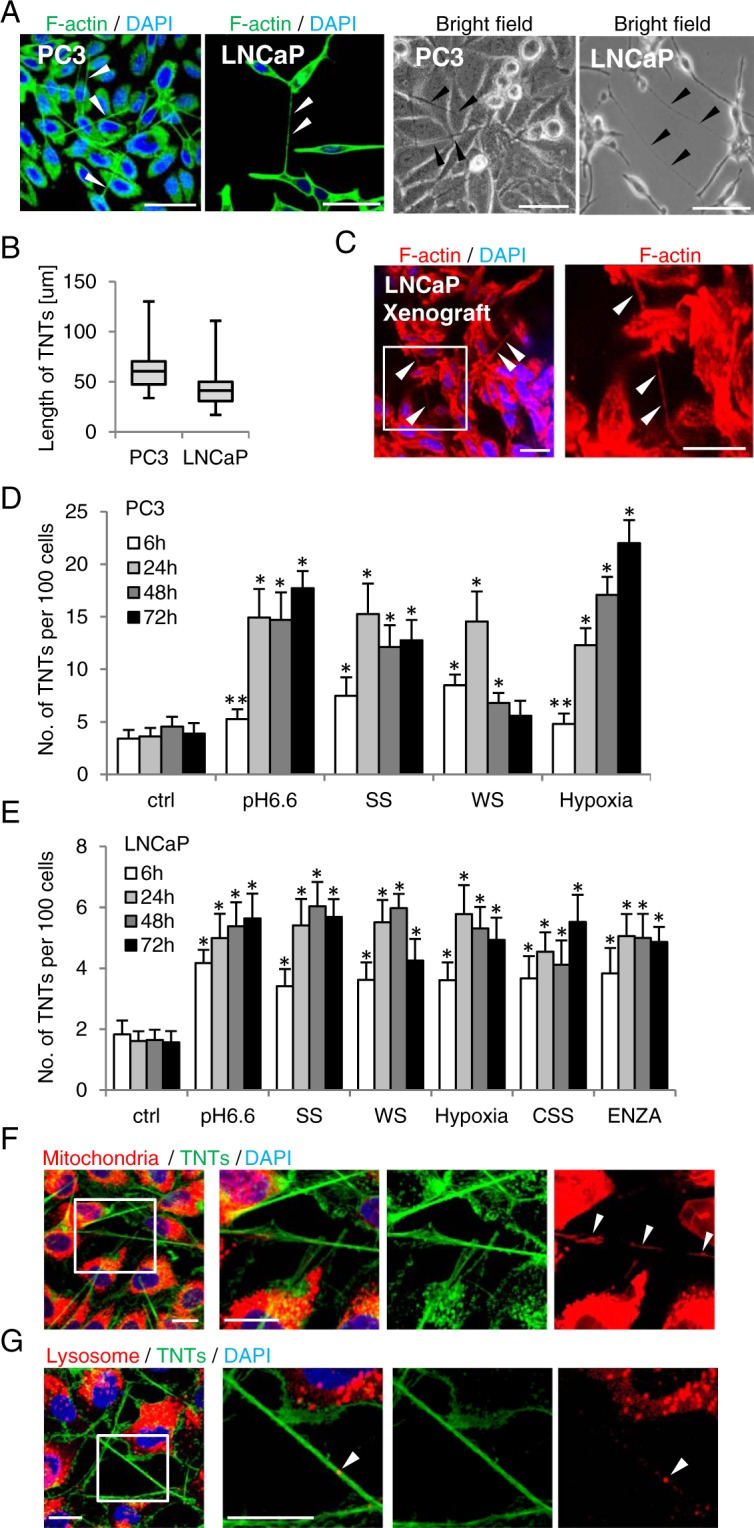
AR blockade and metabolic stresses induce TNTs in PCa cells. (**A**) Exponentially growing PC3 and LNCaP cells were fixed and stained with Alexa Fluor 488-labeled phalloidin (for F-actin) to visualize TNTs under fluorescent confocal microscope or under the bright field (40x lens). Arrows indicate TNTs. (**B**) Lengths of TNTs in PC3 and LNCaP cells were measured and presented as average +/− standard deviation (SD). (**C**) LNCaP xenografts were stained with Alexa Fluor 594-labbeled phalloidin. Right panel is cropped from the white box of the left panel. (**D**) PC3 and (**E**) LNCaP cells receiving stress treatments were stained for TNTs as in (**A**). TNTs were quantified as described in Methods. *p < 0.001 vs. ctrl of the respective group, **p = 0.01 vs. ctrl. Data is presented as average +/− SD from 3 independent experiments. (**F**) LNCaP cells labeled with MitoTracker Deep Red FM (for mitochondria) were treated with 10 μM ENZA for 24 hours and then stained with phalloidin. Co-localization of mitochondria and TNTs was captured by confocal microscopy. Right panel is cropped from the white box of the left panel. (**G**) LNCaP cells treated with 10 μM ENZA for 24 hours were stained for phalloidin and lysosome protein LAMP1. Co-localization of lysosome and TNTs was captured by confocal microscopy. Right panel is cropped from the white frame from the left panel. All scale bars: 20 μm.

We first set out to detect TNTs in PCa cells using immunofluorescence staining for filamentous actin (F-actin) with Alexa Fluor 488-labeled phalloidin in PC3 and LNCaP cells. TNTs were observed in both cell lines under fluorescent microscopy as well as with bright field imaging (Fig. 1A). TNT lengths varied from 33.7 to 130.1 μm in PC3 (median 60.6, interquartile range 47.4–70.3) and 16.9 to 110.8 μm in LNCaP cells (41.1, 30.7–49.9) (Fig. 1B), consistent with prior reports in other cell lines^31,35^. TNTs were also detected in LNCaP xenograft tumor tissue (Fig. 1C).

As progressing cancer cells encounter stress from metabolic demands and tissue hypoxia^36,37^, we evaluated whether varied cellular stresses affect TNT formation. PC3 and LNCaP cells were stressed by acidic microenvironment (pH6.6), serum starvation (SS), whole nutrient starvation with HBSS/HEPES (WS), or hypoxia (1% O_2_) (Fig. 1D,E). AR positive LNCaP cells were also subjected to androgen deprivation using charcoal stripped-serum (CSS, 10%) or the AR-antagonist enzalutamide (ENZA, 10 μM) (Fig. 1E). All stressed conditions significantly induced TNT formation in both cell lines (Fig. 1D,E), including AR pathway inhibition in LNCaP cells (Fig. 1E). Interestingly, however, the microtubule inhibitor, paclitaxel, did not induce TNT formation (Supplementary Fig. S1A). DU145 cells are deficient in gap junctions^38^ but have intercellular bridge structures that play prominent roles in transferring chemical signals^39^. Phalloidin staining in DU145 cells after SS detected TNT-like structures (Supplementary Fig. S1B); however, they are shorter and attached to the substratum, and are therefore distinct from typical TNT morphology^21,31,34^. Consistent with prior reports^26,30^, we detected mitochondria (Fig. 1F; Supplementary Fig. S1C) and lysosomes (Fig. 1G) in TNTs of ENZA-treated LNCaP cells.

We next examined if AR blockade and metabolic stress induce TNT formation in normal prostate epithelial cell line RWPE-1 and the fibroblast osteoblast cell line hFOB. As shown in Figs S1D and S1E, basal levels of TNTs are observed in untreated RWPE-1 and hFOB cells; however, TNT formation was not induced under any of the stressed conditions. Instead, actin-based stress fibres were dramatically induced upon SS and CSS treatments in RWPE-1 (Supplementary Fig. S1F), suggesting different roles for actin-based stress responses in normal compared to cancer cells.

Two models have been proposed for TNT formation, one based on actin-driven protrusion of a TNT structure from one cell towards a target cell, and the other on membrane fusion following intercellular contact, with TNT emergence adherent cell dislodge^40,41^. Live imaging of CSS-treated LNCaP cells is consistent with the latter model, illustrating TNT formation while one cell separated from an adjacent cell (Supplementary Movie S1).

### TNT formation between PCa and osteoblast cells increase after stress

**Figure 2:**
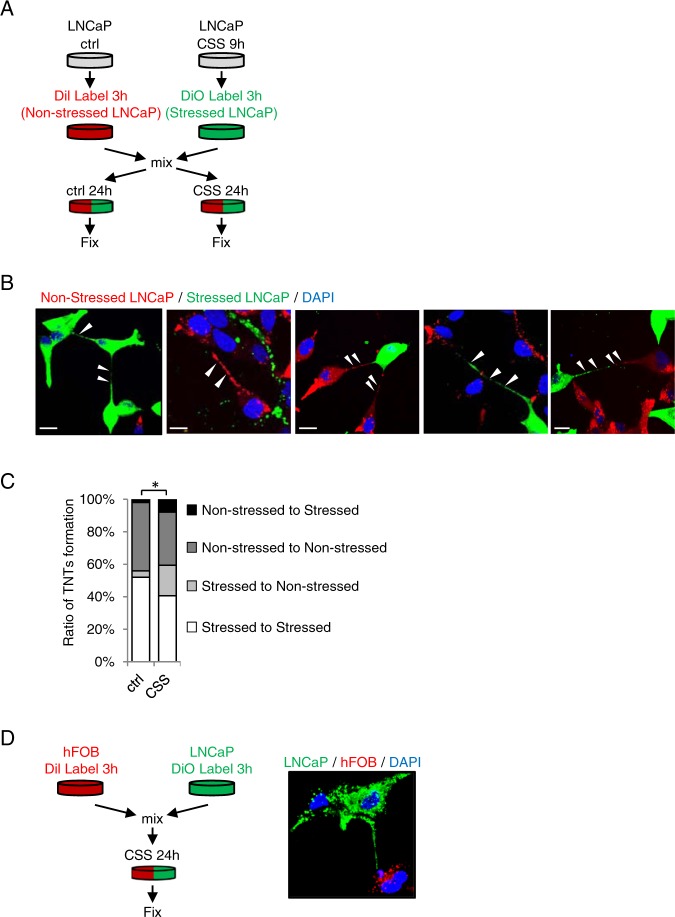
TNTs form between stressed and non-stressed cells. (**A**) Schema illustrating co-culture assays for stressed and non-stressed LNCaP cells. (**B**) Images of TNT formation between co-cultured populations: stressed to stressed cells (green to green), non-stressed to non-stressed cells (red to red), non-stressed to stressed cells (red to green) and stressed to non-stressed cells (green to red). (**C**) TNT formation between co-cultured populations was quantified; percentage of each category is shown. *p < 0.05. (**D**) DiO-labeled LNCaP cells (green) and Dil-labelled hFOB cells (red) were co-cultured in CSS-containing medium for 24 hours. Image shows a TNT from a LNCaP cell (green) towards an osteoblast cell (red). Scale bar: 20 µm.

To further elucidate association of TNT formation and stress responses in PCa cells, various co-culture assays were performed. As illustrated in Fig. 2A, LNCaP cells grown in CSS were labeled with a lipophilic green fluorescent dye DiO (defined as “stressed LNCaP”) while non-treated LNCaP cells were labeled with the red dye, Dil (“non-stressed LNCaP”). Subsequently, the two populations were mixed in a 1:1 ratio and co-cultured under stressed (CSS) or non-stressed (ctrl) conditions for 24 hours. Interestingly, TNTs formed between stressed to stressed cells (green to green), non-stressed to non-stressed cells (red to red), stressed to non-stressed cells (green to red), and non-stressed to stressed cells (red to green) (Fig. 2B). However, in CSS, TNT formation was mostly enhanced from stressed to non-stressed cells as well as from non-stressed to stressed cells (Fig. 2C), indicating preferential TNT communication between stressed and non-stressed populations under these conditions.

Since PCa preferentially metastasizes to bone as osteoblastic lesions^42^, we next modeled PCa-bone microenvironment interactions with co-cultures to assess TNT formation between labelled LNCaP cells and osteoblast hFOB cells using green dye DiO or red dye DiI, respectively. Cells were mixed 1:1 and co-cultured under stressed (CSS) or non-stressed (ctrl) conditions. We detected TNT formation between LNCaP cells as in Fig. 2B, but also from LNCaP to hFOB cells (Fig. 2D). However, TNTs arising from hFOB to LNCaP cells were not observed, suggesting that stressed PCa cells are the driving factors leading the TNT-mediated communication in the bone microenvironment.

### Disruption of TNT formation reduces cancer cell survival

**Figure 3:**
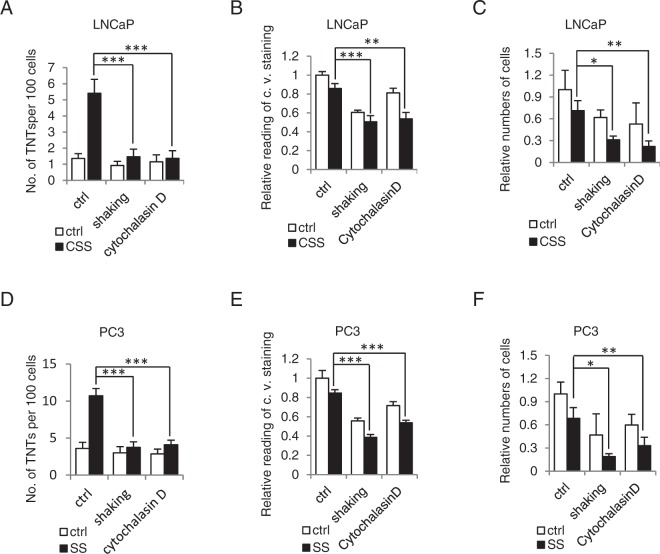
Disruption of TNT formation sensitizes PCa cells to AR blockade and metabolic stress. (**A**) LNCaP cells were treated with androgen deprivation (CSS) combined with TNT disruption by gentle shaking (250 rpm) or actin inhibitor Cytochalasin D and TNT formation was quantified. ***p < 0.001. (**B**,**C**) Cell survival of LNCaP cells after combined treatment with CSS and TNT-disruption was assessed by crystal violate (c.v.) staining (**B**, **p = 0.001, ***p < 0.001) or automated cell counter (**C**, *p = 0.024, ** p = 0.005). (**D**,**E**,F) PC3 cells were treated with SS in combination with TNT-disruption, and TNT formation and cell survival estimated as described for LNCaP cells. *p = 0.002 for shaking, and **p = 0.007 for Cytochalasin D. ***p < 0.001 compared to ctrl. All data is presented as average +/− SD from 3 independent experiments.

To evaluate the functional significance of TNT formation under stressed conditions, we tested whether disruption of TNT formation affected PCa cell survival. Although specific inhibitors on TNT formation have not yet been described, either chemical disruption using actin inhibitors such as Cytochalasin D or mechanical disruption using an automated shaker (250 rpm) are currently well documented approaches to repress TNTs formation^43^. LNCaP cells stressed by CSS were either exposed to Cytochalasin D or automated shaking, and TNT formation and cell survival status were quantified. Both shaking or Cytochalasin D disrupted TNT formation upon CSS treatment, as confirmed via F-actin immunofluorescence staining (Fig. 3A). Disruption of TNT formation significantly reduced cell survival as measured by crystal violet staining (Fig. 3B) or automated cell counter (Fig. 3C) assays. Similar results were observed in PC3 cells, where both chemical-and mechanical-mediated repression of TNT formation reduced PC3 cell survival after serum starvation (Fig. 3D–F).

### Stress chaperone proteins co-localize within and are transported via TNTs

**Figure 4:**
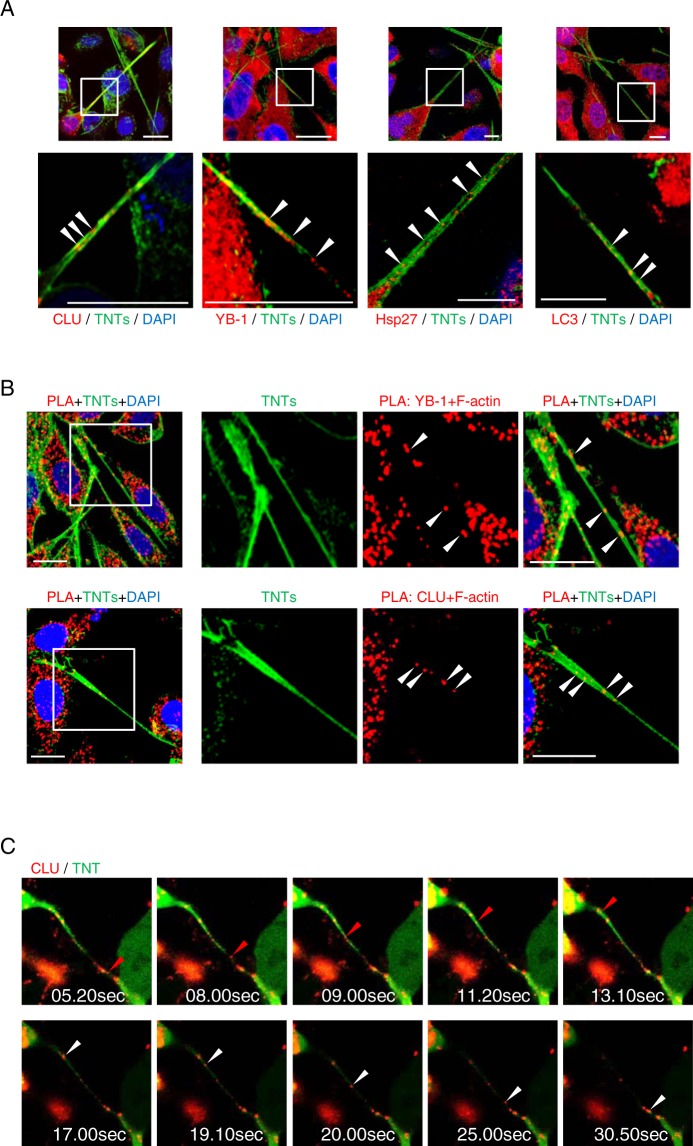
Stress-associated proteins co-localize with and are transported via TNTs. (**A**) PC3 cells treated with SS for 24 hours were co-stained for TNTs (F-actin) and CLU, YB-1, Hsp27 or LC3. Images were taken using confocal fluorescent microscopy (60x). The bottom panel is cropped from the white box of the top panel. (**B**) PLA staining for F-actin and the respective stress-associated proteins (red) was combined with TNT staining (Phalloidin, green) in SS-treated PC3 cells. The right panel is cropped from the white box of the left images. (**C**) LNCaP cells were co-transfected with plasmids expressing mCherry-CLU and eGFP and treated in CSS for 24 hours. Live imaging assay was performed to capture CLU transport along TNTs. Still images created from live cell imaging are shown. In the top panel, red arrows indicate movement of a CLU-positive particle (red) along the TNT (green) from the bottom cell to the top cell. In the bottom panel, white arrows highlight movement of a CLU-positive particle transported in the opposite direction from the top cell to the bottom one. All scale bars: 20 µm.

The preceding data link TNT formation to stress adaptation and cell survival. Since stress chaperone proteins like CLU, YB-1 and Hsp27 orchestrate stress response pathways to facilitate cancer cell survival, we hypothesized that TNT formation may either be regulated by these chaperones, or play roles in transporting stress chaperones between cells^44^. Using immunofluorescence double staining for TNTs (Alexa Flour 488-labelled phalloidin) and the respective protein of interest, we found CLU, YB-1, and Hsp27, as well as autophagosome-related protein LC3, localized within TNTs (Fig. 4A). However, stress granules (stained with G3BP1 or YB-1 antibody), representing another type of stress adaptation structure, were not observed within TNTs after stress granule-induction using arsenite^11^ (white arrows, Supplementary Fig. S2A), even though G3BP1 and YB-1 proteins localized within TNTs (yellow arrows, Supplementary Fig. S2A). A negative control of staining against a cytoplasmic protein vinculin is included in Fig. S2B, which did not show localization of vinculin in TNTs.

Proximity ligation assays (PLA) using antibodies for F-actin and the respective stress-associated proteins of interest with TNT immunofluorescence staining (using Alexa Fluor 488-labeled phalloidin) further confirmed localization of stress chaperone proteins within TNTs in stressed PCa cells. Co-localization (red staining of PLA) of F-actin with CLU, YB-1 (Fig. 4B) or Hsp27 (Supplementary Fig. S2C) were observed in TNTs stained with phalloidin (in green), supporting previous observations of co-localization of CLU, YB-1, or Hsp27 within TNTs shown in Fig. 4A.

To evaluate if these chaperones are transported via TNTs, LNCaP cells were co-transfected with plasmids expressing mCherry-tagged CLU protein and eGFP, and then exposed to CSS for 24 hours. Live cell imaging confirmed that mCherry-CLU is bi-directionally transported via TNTs (Supplementary Movie S2). Detailed time lapse images are shown in Fig. 4C. In the top panel, mCherry-tagged CLU protein (red arrow) moved from the lower cell to the upper cell along the TNT (in green), from 5.20 sec to 13.10 sec; while in the bottom panel, CLU protein (white arrow) was transported in opposite direction to the lower cell along the same TNT, from 17.00 sec to 30.50 sec.

### Stress chaperones CLU and YB-1 affect TNT formation via the Phosphoinositide3-kinase (PI3K) pathway

**Figure 5:**
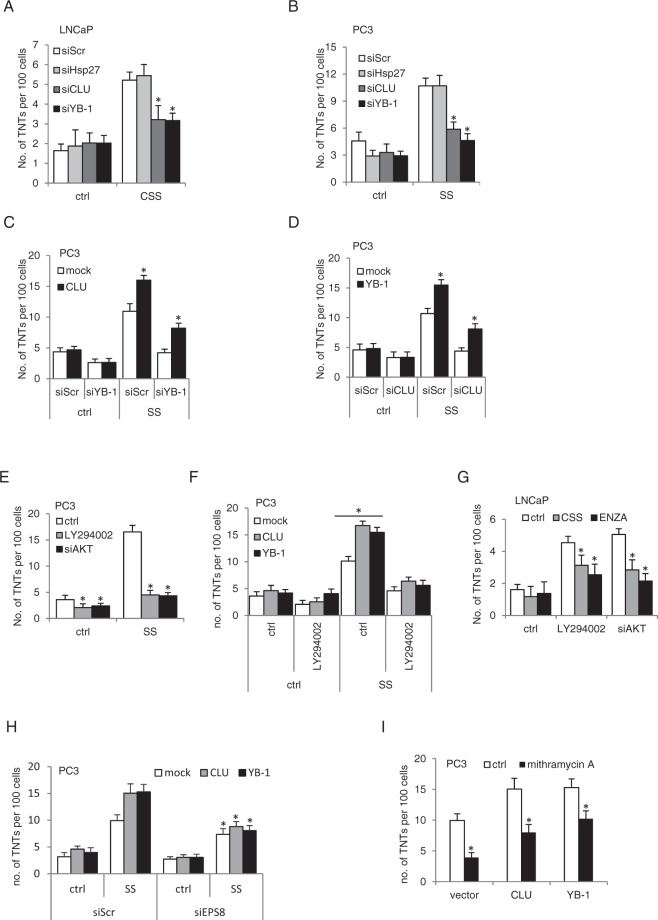
Stress-associated proteins facilitate TNT formation. LNCaP (**A**) and PC3 (**B**) cells were transfected with siRNAs followed with CSS or SS treatment and number of TNTs were quantified. *p < 0.001 compared to siScr. (**C**) PC3 cells were first transfected with siYB-1 and then with CLU-expressing plasmid followed with SS treatment for 24 hours. *p < 0.001 compared to mock. (**D**) PC3 cells were first transfected with siCLU and then with YB-1-expressing plasmid followed with SS treatment for 24 hours. *p < 0.001 compared to mock. (**E**) PC3 cells transfected with siAKT or treated with LY294002 were challenged with SS for 24 hours and then processed for quantification of TNT formation. *p < 0.001 compared to ctrl. (**F**) PC3 cells transfected with vectors for CLU, YB-1 or mock were treated with SS +/− LY294002. *p < 0.001 compared to mock. (**G**) LNCaP cells transfected with siAKT or treated with LY294002 were exposed to CSS or ENZA for 24 hours and then processed for TNT quantification. *p < 0.001 compared to ctrl. (**H**) PC3 cells were transfected with CLU, YB-1 or mock vector; 24 hours later cells were transfected again with siScr or siEsp8, followed with another 24 hours of SS treatment. TNTs formation was quantified. *p < 0.05 compared to SS condiction in the siScr group. (**I**) PC3 cells transfected with CLU, YB-1 or mock vector were challenged with the Eps8 inhibitor mithramycin A in the presence os SS for 24 hours. TNTs formation was quantified. *p < 0.001 compared to ctrl. All data is presented as average +/− SD from 3 independent experiments.

Since CLU and YB-1 have been linked to autophagosome membrane^9^ and stress granule^11^ biogenesis, respectively, we next assessed whether stress chaperone localization in TNTs represent transported cellular cargo or whether they also affect TNT formation. CLU, YB-1 or Hsp27 was silenced in LNCaP and PC3 cells (Supplementary Fig. S3A) which were then stressed by growth in CSS or SS, respectively, for 24 hours. Interestingly, silencing of CLU and YB-1, but not Hsp27, significantly decreased TNT formation under stressed conditions in both LNCaP and PC3 cells (Fig. 5A,B). Similar inhibition of TNT formation was found using the CLU antisense drug OGX-011^45^ (Supplementary Fig. S3B). Gain-of-function studies in PC3 cells transfected with CLU or YB-1 plasmids followed by SS stress showed significant increase of TNT formation under stressed conditions (Supplementary Fig. S3C). CLU and YB-1 silencing similarly modulates TNT formation in MCF7 breast cancer cells under stress conditions (Supplementary Fig. S3D), indicating a broader role of these stress adaptor proteins on TNT formation in other cancers.

To define relative roles of CLU and YB-1 in TNT formation, PC3 cells were silenced with siYB-1 and then transfected with CLU-expressing plasmid. Transfected cells were exposed to SS condition for 24 hours and TNTs were stained and visualized under microscopy. Overexpression of CLU not only enhanced TNT formation by itself, but also rescued siYB-1-repressed TNT formation under SS stress conditions (Fig. 5C). The same effect was observed under low pH stress conditions (Supplementary Fig. S3E). Overexpression of YB-1 also rescued siCLU-repressed TNT formation (Fig. 5D). Collectively, these results imply that either CLU or YB-1 increases TNTs formation under stress conditions.

Since the PI3K pathway has been reported to regulate TNT formation in astrocytes^46^, and since CLU^14^ (Supplementary Fig. S3F) and YB-1 (Supplementary Fig. S3F) positively correlate with pAKT activity, we next defined relationships between PI3K and these stress chaperones in TNT formation in PCa. PC3 cells treated with SS in combination with the PI3K pathway inhibition using siAKT or the inhibitors, LY294002 or wortmannin, significantly reduced TNT formation (Fig. 5E; Supplementary Fig. S3G), and this repression was not rescued by overexpression of CLU or YB-1 (Fig. 5F). This suggests that CLU and YB-1 are upstream regulators of TNT biogenesis that rely on PI3K pathway activity to facilitate TNT formation.

As AR antagonism induces TNT formation in AR-dependent LNCaP cells (Fig. 1E) and also leads to cross-talk activation of P13K/AKT signaling^47,48^, we explored if PI3K/AKT signaling mediates TNT formation after AR blockade. Inhibition of the PI3K pathway with siAKT or an inhibitor LY294002 signficantly suppressed TNT formation in LNCaP cells after CSS or ENZA, indicating that PI3K/Akt signaling supports TNT formation after AR blockade (Fig. 5G).

Epidermal growth factor receptor pathway substrate 8 (Eps8) helps regulate actin remodeling and cytoskeleton reorganization by either direct binding to actin or via complexing with other proteins to activate Rac-associated actin polymerization^49,50^. Eps8 facilitates TNT formation in neuronal cell while inhibiting formation of filopodia^51^. Interestingly, PI3K subunit p85 interacts with the Eps8-containing macromolecular complex to regulate actin remodeling^52^. Eps8 is a key mediator of TNT formation downstream of stress chaperone-induced TNT induction, as Eps8 inhibition by either siRNA or mithramycin A partially repressed CLU- and YB-1- induced TNT formation (Fig. 5H,I), suggesting that CLU or YB-1 may recruit Eps8 and the PI3K/AKT axis to trigger TNT formation.

### AR variant protein is involved in stress-induced TNT formation

**Figure 6:**
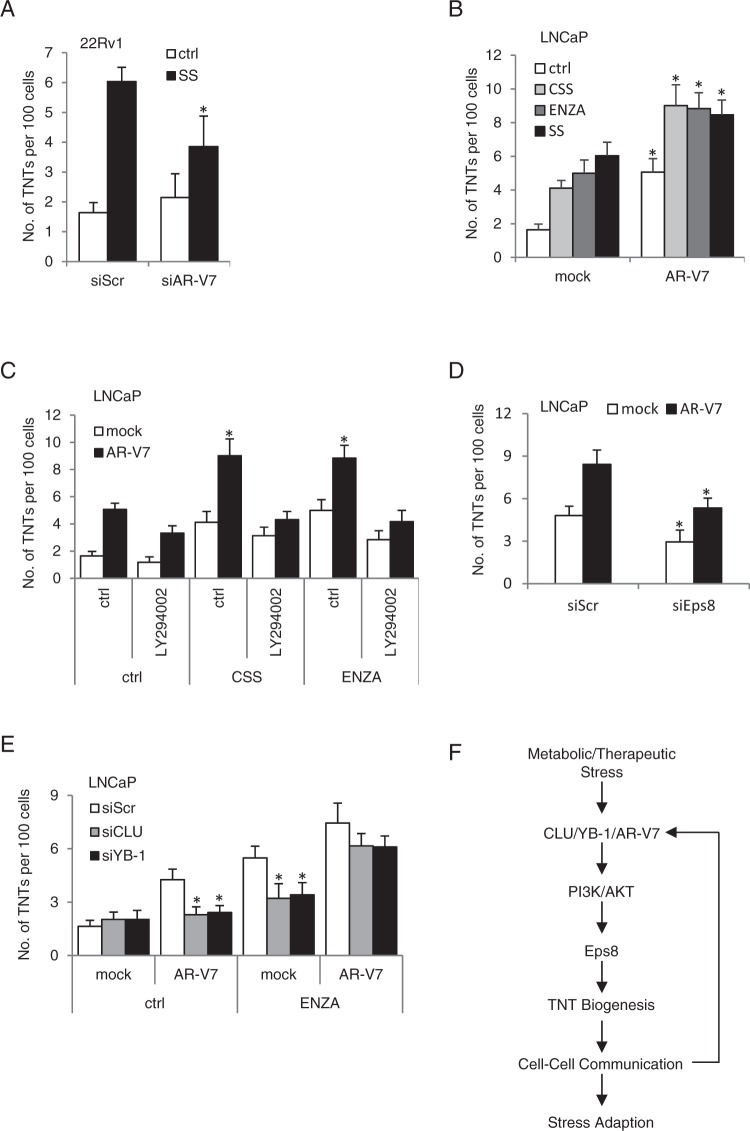
AR variant protein is involved in stress-induced TNTs formation. (**A**) 22Rv1 cells were transfected with siAR-V7 followed with treatment with SS. *p < 0.001 compared to siScr. (**B**) AR-V7 was overexpressed in LNCaP cells and TNT formation was quantified after CSS, ENZA or SS treatments. *p < 0.001 compared to mock. (**C**) LNCaP cells were transfected with AR-V7-expressing plasmid and then treated with CSS or ENZA +/− LY294002 for 24 hours. *p < 0.001 compared to mock. (**D**) LNCaP cells were transfected with plasmids for mock vector or AR-V7, and 24 hours later cells were transfected with siScr or siEps8. Cells were then treated with ENZA for 24 hours and TNTs formation was quantified. *p < 0.001 compared to siScr. (**E**) After CLU or YB-1 silencing in LNCaP cells, AR-V7 plasmid was transfected into the cells, followed with ENZA treatment. *p < 0.001 compared to mock. (**F**) A schema illustrating how ARPI stress induction of CLU/YB-1/AR-V7, via PI3K/AKT modulates Eps8-facilitated TNT production and intercellular communication for cancer cell survival under metabolic and therapeutic stress. All data is presented as average +/− SD from 3 independent experiments.

In addition to cross-talk activation of AKT, ENZA can also induce expression of truncated AR variants lacking the ligand-binding domain that are constitutively active and contribute to castration resistance through activation of a transcriptional signature with distinct gene sets from that of AR full length. AR-variant 7 (AR-V7) was selectively silenced using siRNA in 22Rv1 cells (Supplementary Fig. S4A), which express high levels of functionally active truncated AR variants^53^, followed with SS treatment, to assess their affect on TNT formation during a stress response. Interestingly, AR-V7 silencing suppressed TNT formation under stressed conditions in 22Rv1 cells (Fig. 6A), while AR-V7 overexpression (confirmed by western blotting, Supplementary Fig. S4B) significantly enhanced TNT formation after AR blockade or metabolic stress in LNCaP cells (Fig. 6B).

Since crosstalk interactions occur between AR and PI3K pathways^54^, we next investigated if PI3K contributes to AR-V7 promoted TNT formation. Similar to observations with CLU and YB-1 (Fig. 5F,G), PI3K inhibition with LY294002 attenuated AR-V7-triggered TNT formation (Fig. 6C), indicating that AR-V7 functions upstream of PI3K for TNT formation. This is further confirmed by observations that AR-V7 regulates AKT activity in stressed PCa cells (Supplementary Fig. S4A), identifying a mechanism by which AR-V7 induce TNT formation under stress. Similar to its role in stress chaperone-induced TNT formation, Eps8 also enhances AR-V7-PI3K-induced TNT formation, since Eps8 silencing significantly reduced TNT induction by AR-V7 under ENZA-treated stress (Fig. 6D).

As CLU, YB-1, and AR-V7 all affect TNT formation, we tested whether AR-V7 can rescue siCLU or siYB-1-repressed TNT formation. LNCaP cells were transfected with siCLU or siYB-1, followed by overexpression of AR-V7 and ENZA treatment. Interestingly, AR-V7 rescued siCLU- or siYB-1-repressed TNT formation only after ENZA (Fig. 6E), suggesting a potential context-dependent role for AR-variants in TNT formation as part of a stress response when AR full length protein is antagonized. Our data link stress chaperone induction with PI3K/AKT signaling activation and Eps8-mediated actin remodeling that orchestrate TNT formation and intercellular communication for the survival of cancer cells under stress (Fig. 6F).

## Discussion

TNTs are recently described structures for long-distance intercellular communication and are consequently involved in many cellular pathways including signal transduction, nanoparticle delivery, immune responses, embryogenesis, apoptosis, cell differentiation and reprogramming, as well as cancer initiation and progression^17,46,55^. TNT formation is induced by cellular stressors like nutrient deprivation, low pH or hypoxia^34,56^. This work provides the first characterization of TNT formation in PCa as a means for stress adaptation and survival in the context of ARPI. ARPI induces ER stress, autophagy and activation of stress response pathways involving molecular chaperones, kinase and transcriptional signalling, and alternative splicing^5,13,44,57^. The detection of TNT induction after AR inhibition identifies another mechanism of stress-driven communication and material exchange processes. Interestingly, ARPI induces TNT formation in PCa, but not in normal prostatic epithelial cells, suggesting that TNTs are adaptive response specifically coopted by PCa cells to survive therapeutic stress. This is further supported by findings that inhibition of TNT formation reduce cell survival under stress (Fig. 3). Moreover and of particular relevance to the bone microenvironment and site-specific osseous metastases typical of PCa, PCa cells produce TNTs to connect to osteoblasts, which may be used for heterocellular transportation and communication^30,42^. Further investigations will focus on stromal specific induction of TNT and relevance to bone microenvironment and PCa metastases.

In addition to identifying TNT’s arising from stressed PCa cells to neighbouring unstressed cancer and bone stromal cells, this study also characterized transport and role of stress chaperones associated with treatment resistance in PCa. Castration-related activation of stress chaperones YB-1, Hsp27 and CLU are linked to a variety of pro-survival mechanisms that mediate CRPC^13,44^. We found these stress chaperones all locate within TNTs. Interestingly, live cell imaging (Supplementary Movie S2) illustrated bi-directional CLU-containing particle transport via a TNT connecting two LNCaP cells. This may reflect exchange of differently modified CLU proteins or varied CLU-containing organelles between cells. CLU traffics under stress to mitochondria to suppress Bax activation and apoptosis^58^, and also facilitates Atg-3 mediated LC3 lipidation during autophagosome biogenesis^9^. Both mitochondria and autophagosomes are transported via TNT^30,59^, hence bi-directional transport of CLU-containing particles implicates transport of CLU-containing mitochondria or autophagosomes from one cell to another under stressed conditions. Factors regulating transport of specific cellular cargos in specific directions require further investigation.

The functional role of stress adaptor proteins in TNT formation is undefined. While this study identifies that YB-1, Hsp27 and CLU all localize in TNTs, only CLU and YB-1 positively regulate TNT biogenesis in cancer cells. ENZA-induced stress leads to YB-1 and CLU mediated activation of MAPK and AKT^60^, which may be one mechanism promoting TNT formation. YB-1 or CLU appear to be upstream mediators of a key role for PI3K/AKT activation in TNT biogenesis, as their overexpression did not rescue TNT formation when PI3K/AKT pathway is inhibited (Fig. 5F,G). Surprisingly, Hsp27, which regulates F-actin filament dynamics^61^ and AKT signalling^44^ did not alter TNT formation. If the complex roles of Hsp27 on regulating actin microfilaments and polymerization are involved with TNT formation requires further exploration. PI3K subunit p85 interacts with the Eps8-containing protein complex to regulate actin polymerization^52^. Silencing of Eps8 attenuated stress-induced TNT-induction (Fig. 5H,I), further defining a novel mechanistic network of stress chaperones, PI3K/AKT signaling and actin remodeling for TNT induction that supports cell communication and survival under stressed conditions.

AR-variants are induced under the selective pressure of AR pathway inhibition, and are associated with resistance to AR pathway inhibitors and poor survival in CRPC^62,63^. These variants transcriptionally regulate sets of genes distinct from AR full length, and we found that AR-V7 can induce TNT formation and also rescue siCLU- or siYB-1-repressed TNT formation during ENZA treatment stress. This identifies a potential mechanism by which AR-variants can support TNT formation during treatment stress with AR antagonists. While YB-1 has been implicated in regulating AR-V7 expression in LNCaP cells^64^, in the current study, YB-1 silencing did not affect AR-V7-induced TNT formation post ENZA (Fig. 6E). These data support a model whereby AR antagonism leads to activation of CLU, YB-1, and AR-variants that facilitate PI3K-Eps8-mediated TNT formation and intercellular communication during treatment stess.

In summary, TNTs form between stressed and neighboring unstressed PCa cells, as well as with osteoblast cells, to support cell survival under stress conditions. This work defines a mechanistic network involving ARPI induction of chaperone and AR variant proteins, PI3K/AKT signaling, and actin remodeling to support TNT formation that confer stress adaptation and treatment resistance. Since stress chaperones and AR-variants are defined mediators of CRPC, these findings implicate stress-induced TNT formation as a mechanism for intercellular communication, stress adaptation, and treatment resistance in PCa.

## Methods

### Cell lines

Human cancer cell lines PC3 and 22Rv1 cells were acquired from the ATCC. LNCaP cells were kindly provided by Dr. Leland W. K. Chung (Emery University). LNCaP and 22Rv1 cells were maintained in RPMI-1640 media (Invitrogen Life Technologies, Burlington, ON) containing 10% heat inactivated fetal bovine serum (FBS; Invitrogen Life Technologies). For treatment with CSS, cells were grown in 10% CSS (Invitrogen Life Technologies) instead of FBS. PC3 cells and DU145 cells were maintained in Dulbecco’s modified Eagle’s medium (DMEM; Invitrogen Life Technologies) containing 10% FBS. Normal prostatic epithelial RWPE-1 cells were maintained in Keratinocyte Serum Free Medium (Invitrogen-Gibco, Kit Catalog Number 17005–042) supplied with 0.05 mg/ml bovine pituitary extract and 5 ng/ml human recombinant epidermal growth factor (Invitrogen Life Technologies). hFOB osteoblast cells were from ATCC and were maintained in DMEM/F12 1:1 medium (Hyclone, Catalog number SH30272.01) supplied with 10% FBS and 0.3 mg/ml G418. hFOB were grown at 33 °C for passaging and at 37 °C for the co-culture assay with LNCaP cells. MCF7 cells were received from ATCC and cultured with Eagle’s Minimum Essential Medium (ATCC) containing 0.01 mg/ml human recombinant insulin and 10% FBS.

### Reagents and antibodies

LY294002 and wortmannin were purchased from Cell Signaling Technology. CytochalasinD was purchased from Sigma Life Sciences. Enzalutamide was provided by Haoyuan Chemexpress Co. Primary antibodies used in this study were: ALexa Fluor 488 Phalloidin (A12379) and ALexa Fluor 594 Phalloidin (A12381) from Life Technologies; anti-CLU (sc-6419) and anti-AR (sc-7305) from Santa Cruz Biotechnology; anti-F-actin (ab130935) and anti-YB-1 (ab76149) from Abcam, and anti-LC3 (LC3B, #2775) from Cell Signaling Technology. Secondary antibodies for immunofluorescence staining were as follows: donkey anti-rabbit ALexa Fluor 594 (A-21207), donkey anti-goat ALexa Fluor 488 (A-11055) and donkey anti-rabbit ALexa Fluor 488 (A-21206), all from Invitrogen Life Technologies.

### Transfection

For transfection of plasmids, X-treme GENE9 DNA transfection reagent (Roche, Mannheim, Germany) and Lipofectin (Invitrogen Life Technologies) were applied for PC3 and LNCaP (and 22RV1) cells, respectively, according to manufacturer’s user guides. For transfection of siRNA, oligofectamin (Invitrogen Life Technologies) was used according to the user guide. The siRNAs for scramble: 5′-CAGCGCUGACAACAGUUUCAU-3′; CLU: 5′-GCAGCAGAGUCUUCAUCAU-3′; YB-1: 5′-UGACACCAAGGAAGAUGUAUU-3′; siHsp27: 5′-GUCUCAUCGGAUUUUGCAGC-3′; and siAR-V7: 5′- GUAGUUGUGAGUAUCAUGA-3′ were from Dharmacon. siRNA targeting AKT was purchased from Cell Signaling Technolody and siRNA targeting Eps8 was from Santa Cruz.

CLU antisense oligonucleotide OGX-011 was provided by OncoGenex Pharmaceuticals (Vancouver, BC). The sequence of OGX-011 corresponds to the human CLU translation initiation site in exon ll (5′-CAGCAGCAGAGTCTTCATCAT-3′). A scrambled control oligonucleotide (ScrB, 5′-CAGCGCTGACAACAGTTTCAT-3′) was provided by ISIS Pharmaceuticals (Carlsbad, CA).

### Immunofluorescence staining and confocal microscopy

Cells grown on glass coverslips were fixed with 3.5% paraformaldehyde (PFA) and permeabilized with 0.5% Triton X-100. After blocking with 3% skim milk, cells were incubated with primary antibody overnight at 4 °C followed by incubation with secondary antibody at 37 °C. Nuclei were counterstained with 4′,6-diamidino-2-phenylindole (DAPI)-containing mounting medium (VECTOR, Burlingame, CA). For TNT visualization, cells were stained with ALexa Fluor 488-labeled phalloidin for 3 hours at 37 °C. Fluorescence images were obtained using a Zeiss LSM 780 confocal microscope (Carl Zeiss, Thornwood, NY). Images were collected with ZEN software using a 40x or 63× 1.40 oil Plan-Apochromat DIC M27 Zeiss objective with the confocal pinhole set to 1 Airy unit. All fluorescent images for TNTs were taken from the z-stack settings of the ZEN software.

For the proximity ligation assay (PLA) analysis, cells stained with the antibody solution of F-actin/Hsp27, F-actin/YB-1 or F-actin/CLU overnight. Protein-protein interaction was identified using a commercially available PLA kit (Duolink; Sigma-Aldrich) as per manufacturer’s protocol.

To investigate TNTs in tissues, frozen LNCaP xenografts were dissected into 1 mm^3^ cubes, fixed with 3.5% PFA and permeabilized with Triton X-100. Tissues were then incubated with 3% BSA to block unspecific staining and then stained with Alexa Fluor 594-labeled phalloidin for F-actin at 4 °C in dark. Nuclei were counterstained with DAPI solution. Samples were examined under LSM780 confocol microscope and images were captured using a 40× 1.40 oil Plan-Apochromat DIC M27 Zeiss objective with the confocal pinhole set to 1 Airy unit. The images were taken with the z-stack settings.

### Quantification of TNTs

TNTs were identified based on previously published morphologic criteria^17,21,24,31,34,40,56^. Mandatory morphologic features include: no adherence to cell culture plate substratum (cells hover freely or pass over adherent cells); estimated width of the extension 1000 nm or less; and narrow extension base. TNTs and cell numbers were counted by two researchers A.K. and F.Z. in at least 10 randomly chosen visual fields using a 40× 1.40 oil Plan-Apochromat DIC M27 Zeiss objective. There is no difference on the quantification of TNT formation between fixed (wirh 3.5% PFA for 15 min at room temperature) and non-fixed cells in our model (Supplementary Fig. S4C).

### Live cell imaging

Cells grown in glass-bottom dish were cultured in a chamber supplied with 5% CO_2_ at 37 °C with humidity on the spinning disc microscope (Zeiss). Time lapse live images were taken with ZEN software under 40x oil lens for indicated time course.

### Cell survival analysis

For the crystal violet staining, cells were fixed and stained with 0.1% crystal violet for 5 minutes and dried overnight. After one-hour incubation with Soerensen’s solution, relative staining was quantified using Gen5 software (version 1.11) and a BioTek® Epoch plate reader. For cell number counting assay, an automated cell counter (TC-20, Bio Rad) was recruited to count cell numbers of trypsinized cell solution.

### Statistical analysis

Data was analyzed by two-tailed Mann-Whitney-U and Kruskal-Wallis test as well as Chi^2^ test using SPSS V23.0 (IBM, Armonk, NY, USA). Error bars display standard deviations.

## Supplementary information

Supplementary informartion

Supplementary Movie-1

Supplementary Movie-2
