# Supplementary material for: Stress-induced tunneling nanotubes support treatment adaptation in prostate cancer
Source: Sci Rep. 2019 May 24;9:7826. doi: 10.1038/s41598-019-44346-5 (PMC6534589; doi:10.1038/s41598-019-44346-5)

Supplementary Information

Stress-induced tunneling nanotubes support treatment adaptation in prostate cancer

Alexander Kretschmer[1], Fan Zhang[1], Syam Prakash Somasekharan[1], Charan Tse[1], Lauren Leachman[1], Anna Gleave[1], Brian Li[1], Ivan Asmaro[1], Teresa Huang[1], Leszek Kotula[2], Poul H. Sorensen[3], Martin E. Gleave[1]

[1]The Vancouver Prostate Centre, Department of Urological Sciences, University of British Columbia, 2775 Laurel Street, Vancouver, British Columbia, Canada V6H 3Z6

[2]Department of Urology, Biochemistry and Molecular Biology, Medicine SUNY Upstate Medical University, Syracuse, NY 13210

[3]Department of Pathology and Laboratory Medicine, University of British Columbia, Vancouver, British Columbia, Canada, V6H 3Z6

Short title: TNTs support cancer cell survival under stress

Keywords: cellular stress, tunneling nanotubes, prostate cancer, androgen receptor, stress chaperones, treatment resistance

Corresponding Author: Martin E. Gleave, Department of Urologic Sciences, University of British Columbia, 2775 Laurel Street, Level 6, Vancouver, British Columbia, Canada V6H 3Z6. Phone: 604-875-4818; Fax: 604-875-5654; m.gleave@ubc.ca.

Supplementary Information

Supplementary Methods

Western blot analysis. Whole protein lysates were collected using 1x sample buffer (50mM Tris-HCl, pH6.8, 2% SDS, 10% glycerol, 0.1M DTT and 0.02% bromophenol blue) and consequently separated on 10% polyacrylamide gels after boiling. Proteins were transferred to nitrocellulose membranes (BIO-RAD Laboratories, Mississauga, ON) and incubated with Odyssey® Blocking Buffer (LI-COR, Lincoln, NA, USA) to block unspecific signals. Membranes were then incubated with respective primary antibodies for overnight and then the secondary antibodies for one hour. Membranes were scanned using a Li-Cor Odyssey infrared imager and Image Studio Software (version 5.2). Molecular weight markers are included in all blots. Primary antibodies used in the studies are: anti-CLU (sc-6419) and anti-AR (H-280) from Santa Cruz Biotechnology; anti-YB-1 (ab76149) from Abcam, anti-pAKT (#40605) and anti-tAKT (#30635) from Cell Signaling Technology; and anti-vinculin (V9131) from Sigma-Aldrich. Secondary antibodies for western blot: donkey anti-goat IgG (680, #926-68074; 800, #926-32214), donkey anti-mouse IgG (680, #926-68072; 800, #926-32212) and donkey anti-rabbit IgG (680, #926-68073; 800, #926-32213) all purchased from Li-Cor. Original blots before crop are shown in the Supplementary Figure S5.

Supplementary Figure Legends

Supplementary Figure S1. TNTs formation in prostate cells and osteoblast. (A) PC3 cells received 10 µM Paclitaxel for 24 hours were fixed and stained with Alexa Fluor 488-labeled phalloidin (for F-actin). TNTs formation was examined under fluorescent confocal microscope. The numbers of TNTs were shown in the left panel and the representing image was shown in the right panel. Scale bar: 20 µm. (B) PCa DU145 cells were treated with SS and then stained with Alexa Fluor 488-labeled phalloidin and TNTs-like structures are captured with fluorescent

confocal microscope. Right image is cropped and enlarged from the white frame of the left image. Scale bar: 20 µm. (C) LNCaP cells treated with ENZA for 24 hours were fixed and stained with Alexa Fluor 488-labbeled phalloidin and mitochondria marker protein Cytochrom c. Co-localization of mitochondria and TNTs was captured under confocal microscope. Right panel is cropped from the white frame from the left panel. Scale bar: 20 µm. (D) Normal prostate epithelial cell line RWPE-1 was exposed to various stress conditions for 24 hours and TNTs formation was evaluated with F-actin staining. Images of F-actin staining (top panels) and graphs of numbers of TNTs formed (low panels) are shown. White arrow shows typical TNTs. Scale bar: 20 µm. (E) osteoblast hFOB cells were exposed to various stress conditions for 24 hours and TNTs formation was evaluated with F-actin staining. Images of F-actin staining (top panels) and graphs of numbers of TNTs formed (low panels) are shown. White arrow shows typical TNTs. Scale bar: 20 µm. (F) RWPE-1 cells exposed to SS or CSS together with the non-treated (ctrl) were stained with F-actin. Images were taken with confocal microscope. Scale bar: 20 µm. All data is presented as average +/- SD from 3 independent experiments.

Supplementary Figure S2. Localization of stress-associated proteins with TNTs. (A) PC3 cells were exposed to stress granule-inducing agent arsenite (0.5 mM) for one hour and double staining for G3BP1/F-actin or YB-1/F-actin were conducted on the cells. Right panel is the enlarged image from the white frame of the left picture. Yellow arrows show the G3BP1- or YB-1-positive particles localized with the TNT (green); white arrows show the stress granules (red) which are too large to exist within the TNT (green). (B) PC3 cells treated with SS for 24 hours were fixed, and double immunofluorescent staining on F-actin (for TNTs) and vinculin were performed. Images were taken with a confocal fluorescent microscope. Scale bar: 20 µm. (C) PLA staining for F-actin and Hsp27 in combination with immunofluorescence staining for Phalloidin (for TNT) in SS-treated PC3 cells. White arrows indicate the PLA positive staining for F-actin and Hsp27 (red) localizing within TNTs (green). Scale bar: 20 µm.

Supplementary Figure S3. Stress-associated proteins affect TNTs formation. (A) Protein lysates from PC3 cells transfected with siRNAs targeting CLU, YB-1 or Hsp27 were processed to western blot assays to confirm knocking down effect. Vinculin (vin) was used as loading control. (B) CLU was knocked down with either antisense oligonucleotide OGX-011 or siCLU in PC3 cells followed with SS treatment. TNTs formation was evaluated. ***, $p<0.001$ compared to ScrB. (C) CLU- or YB-1-expressing plasmid was transfected into PC3 cells. Cells were then treated with SS for 24 hours and number of TNTs formed as well as the expression levels of CLU and YB-1 proteins were shown. ***, $p<0.001$ compared to mock. (D) MCF7 cells were transfected with siRNAs targeting CLU, YB-1 or Hsp27 and then exposed to 100 µM H_2O_2 for 24 hours. TNTs formation was evaluated. Whole protein lysates were analysed with western blot against proteins of interest. Vinculin (vin) as shown as loading control. *, $p<0.01$ compared to siScr H_2O_2. (E) PC3 cells were firstly transfected with siYB-1, and then transfected with CLU-expressing plasmid followed with low pH treatment (pH6.6) for 24 hours. TNTs formation was evaluated. **, $p=0.005$, ***, $p<0.001$ compared to mock. (F) PC3 cells transfected with siCLU (top panel) or siYB-1 (lower panel) were exposed to SS treatment for 24 hours. Whole protein lysates were collected and processed for western blot against the antibodies listed. (G) PC3 cells were treated with SS in the presence of an inhibitor of PI3K pathway wortmannin and then processed for the investigation of TNTs formation. *, $p<0.05$, ***, $p<0.001$ compared to ctrl. All data is presented as average +/- SD from 3 independent experiments.

Supplementary Figure S4. Androgen signaling axis is involved with TNTs formation. (A) 22Rv1 cells were transfected with siRNA targeting AR-V7 and then treated with SS for 24 hours. Whole protein lysates were collected and processed for western blot. Vinculin (vin) was used as loading control. (B) LNCaP cells were transfected with AR-V7-expressing plasmid or vector plasmid (mock) and then exposed to ENZA for 24 hours. Whole protein lysates were collected and processed for western blot. The AR blot image showing here is cropped from different lanes

from the same blot. (C) Numbers of TNTs were quantified in PC3 cells treated with SS followed with either fixation with 3.5% PFA or no fixation. The data is presented as average +/- SD from 3 independent experiments.

Supplementary Figure S5. Original blots for the western blot results presented in this study.

Supplementary Movie 1. Formation of TNTs in stressed LNCaP cells. LNCaP cells grown in glass-bottom dish were cultured in a chamber supplied with 5% CO_2 at 37 °C with humidity on the spinning disc microscope (Zeiss). Cells were treated with CSS and the time lapse live images were taken with ZEN software under 40x oil lens for indicated time course.

Supplementary Movie 2. Transportation of CLU-positive particles along a TNT. LNCaP cells grown in glass-bottom dish were co-transfected with mCherry-CLU and eGFP plasmids. 24 hours after transfection cells were cultured in a chamber supplied with 5% CO_2 at 37 °C with humidity on the spinning disc microscope (Zeiss) and received CSS treatment for another 24 hours. Live imaging assay was carried out using ZEN software to capture the transportation of CLU protein along the TNTs.

Supplementary Figure 1

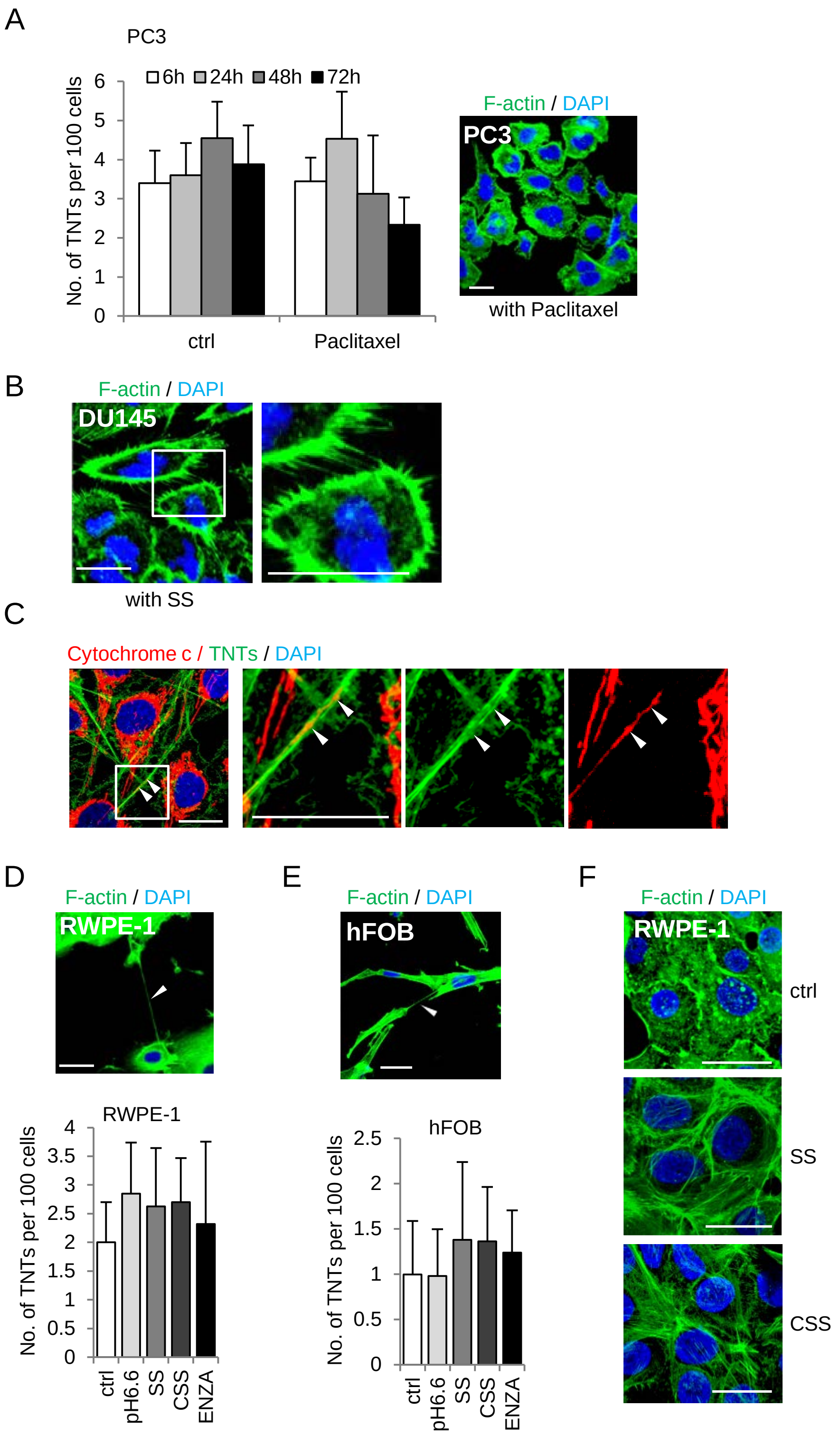

Supplementary Figure 2

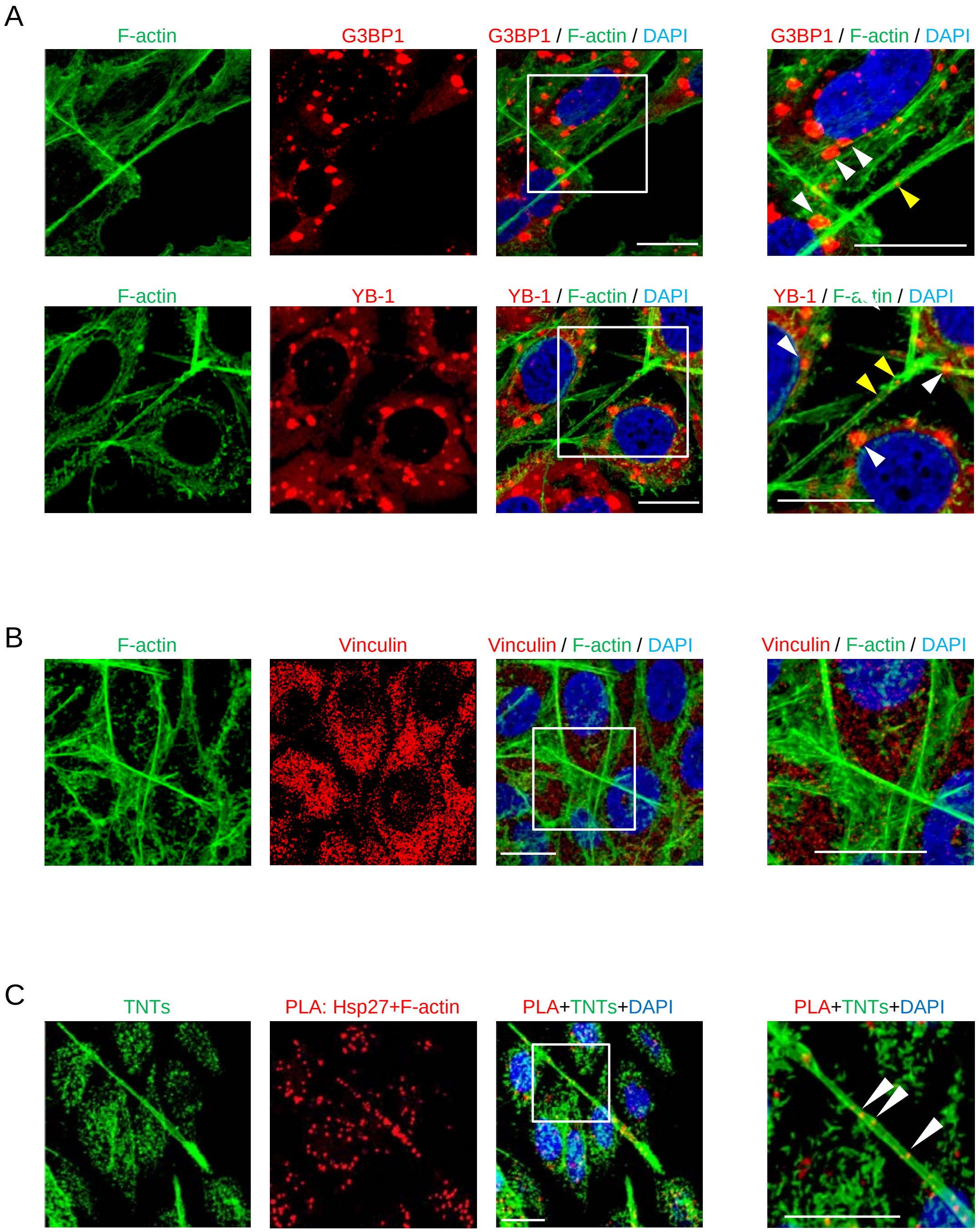

Supplementary Figure 3

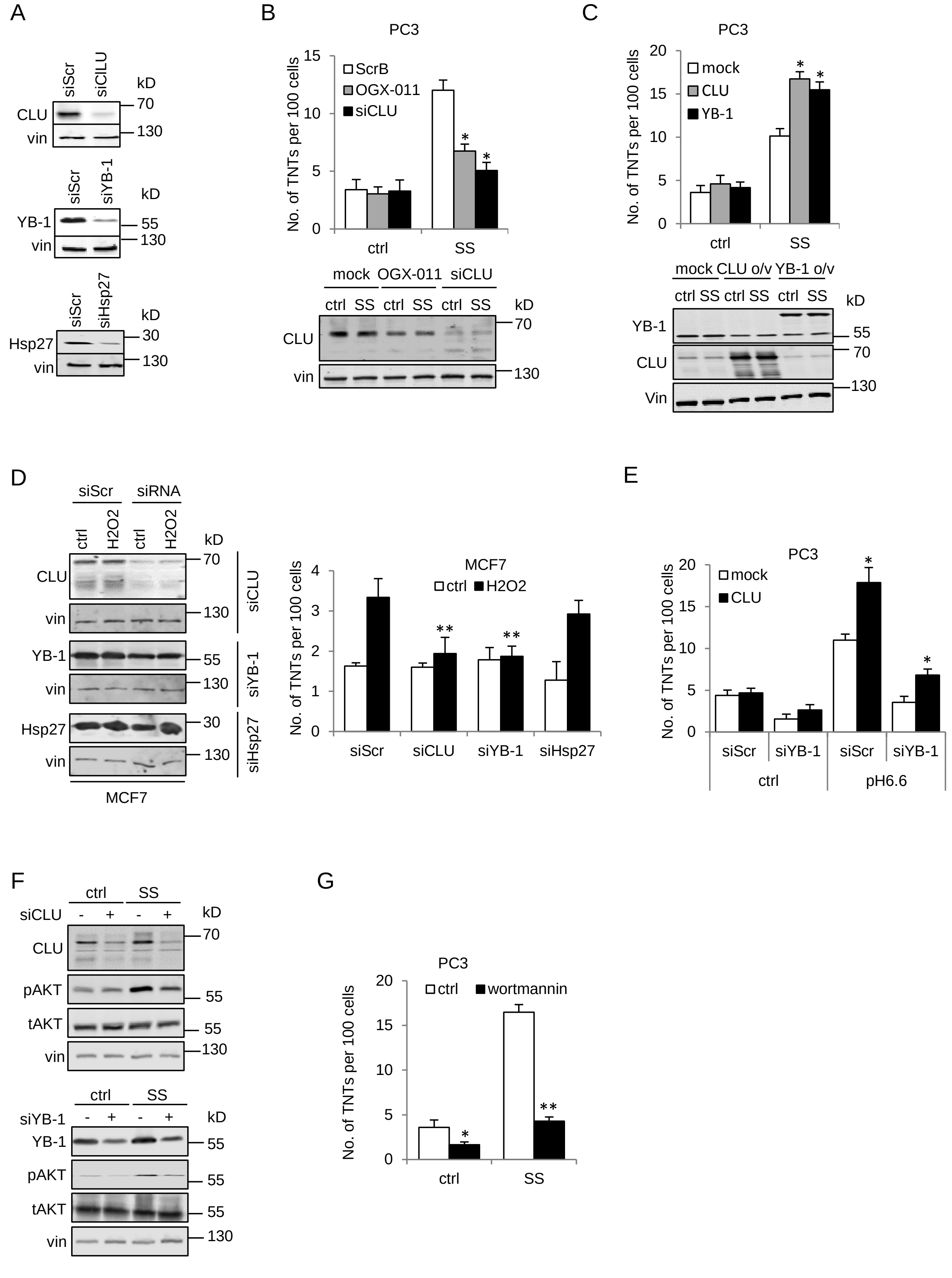

Supplementary Figure 4

A

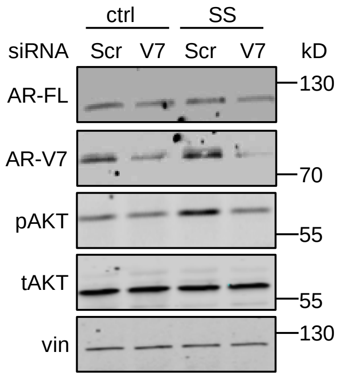

B

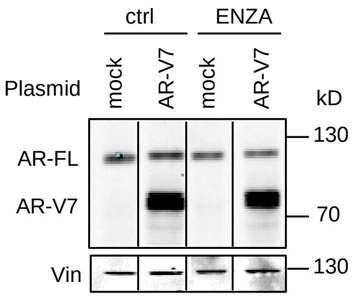

C

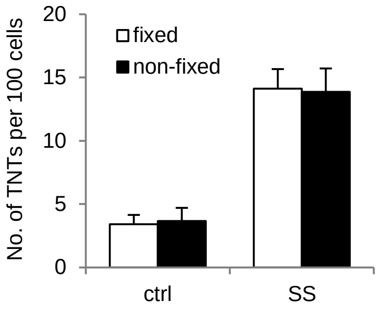

Supplementary Figure 5

Fig. S3A Original blots

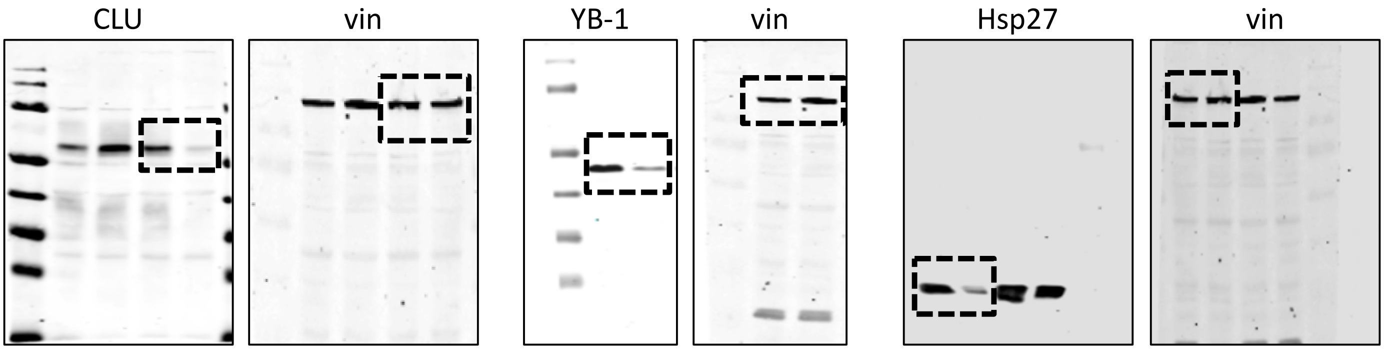

Fig. S3B Original blots

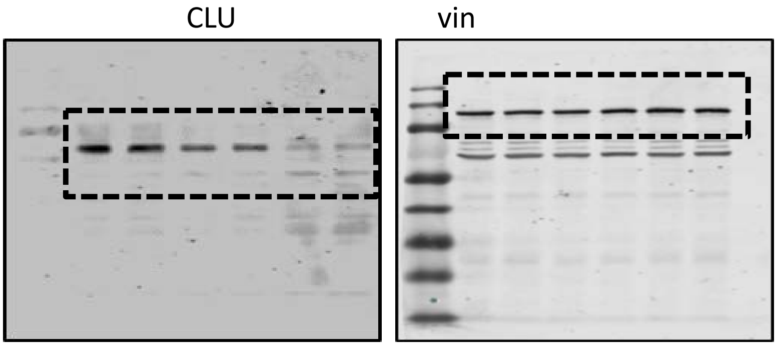

Fig. S3C Original blots

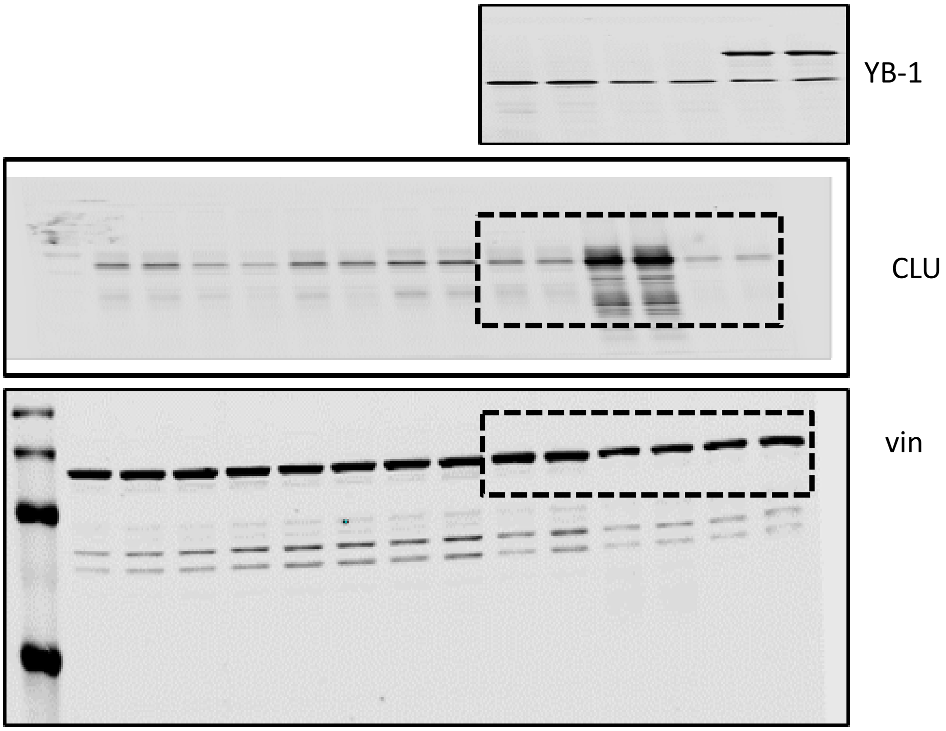

Fig. S3D Original blots

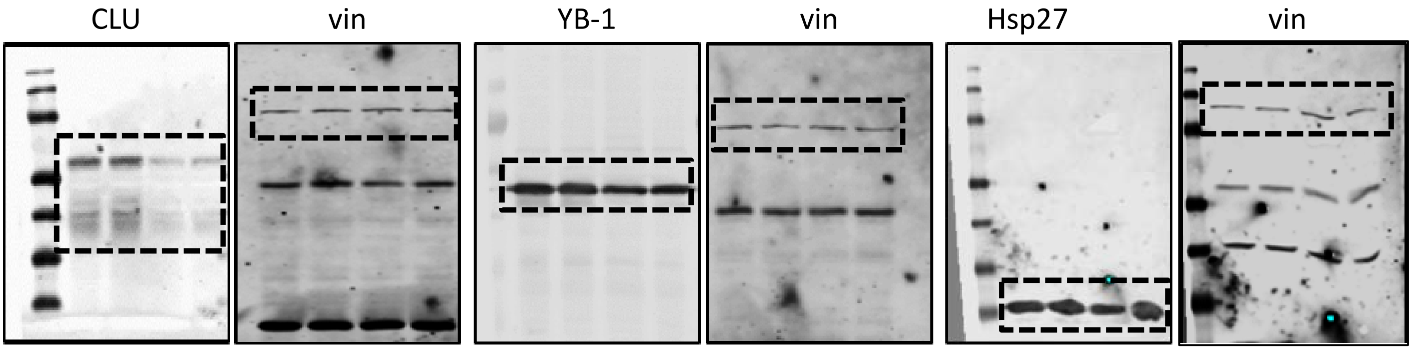

Fig. S3F Original blots

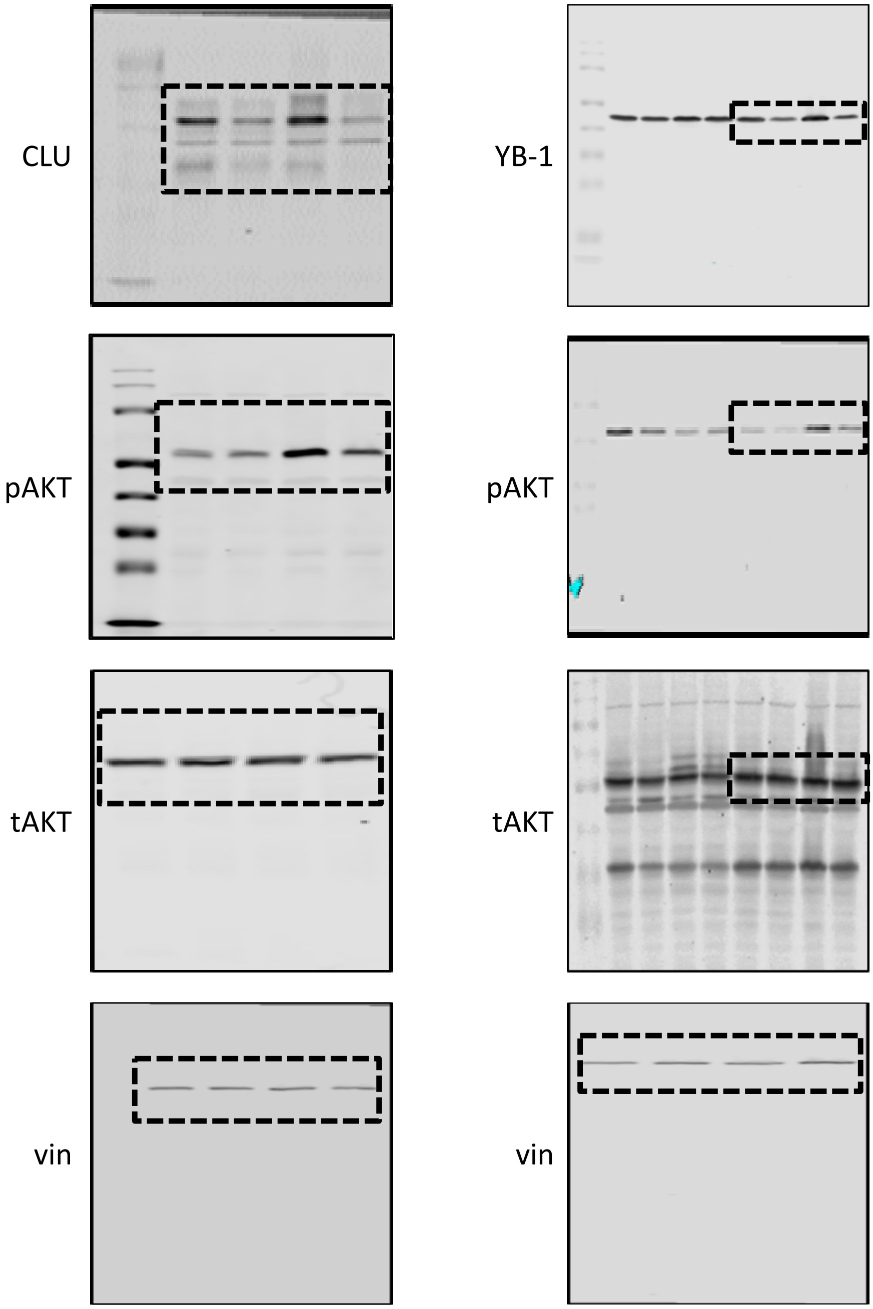

Fig. S4A Original blots

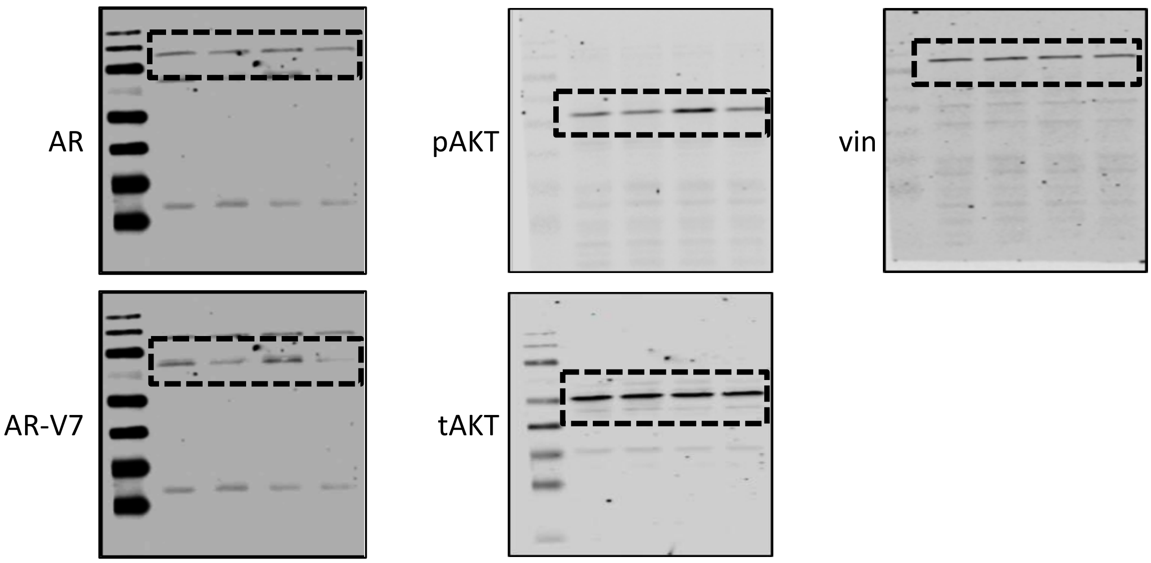

Fig. S4B Original blots (lanes 1, 3, 7, 9)

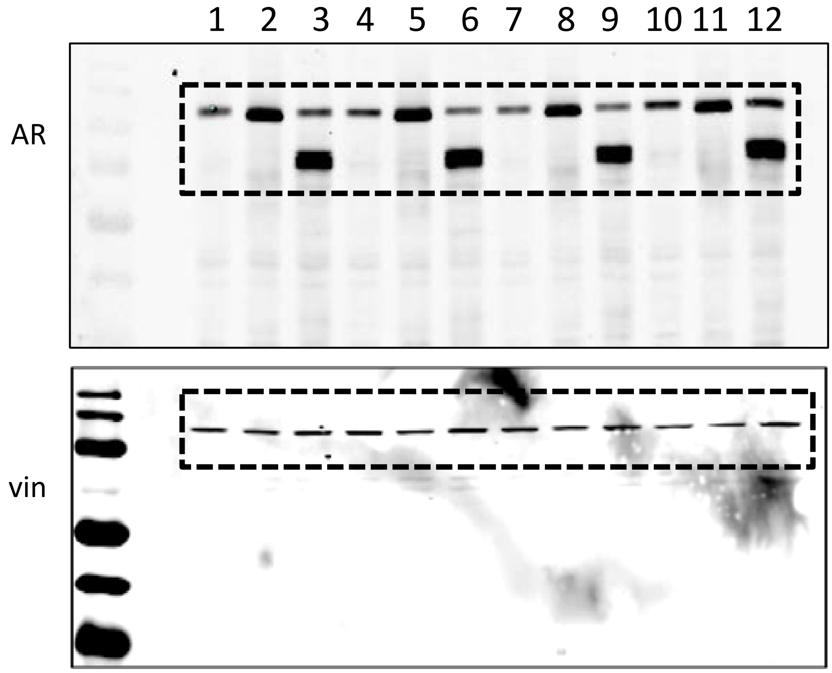

Supplement: Supplementary file 1 — Supplementary informartion [file 41598_2019_44346_MOESM1_ESM.pdf]
